# IgG Antibodies to GlcNAc*β* and Asialo-GM2 (GA2) Glycans as Potential Markers of Liver Damage in Chronic Hepatitis C and the Efficacy of Antiviral Treatment

**DOI:** 10.1155/2018/4639805

**Published:** 2018-12-02

**Authors:** Eugeniy Smorodin, Boris Sergeyev, Oleg Kurtenkov, Tatiana Kuznetsova, Julia Geller

**Affiliations:** ^1^Department of Oncology & Immunology, National Institute for Health Development, Hiiu 42, 11619 Tallinn, Estonia; ^2^Department of Virology, National Institute for Health Development, Hiiu 42, 11619 Tallinn, Estonia

## Abstract

Total serum IgG level is a surrogate marker of hepatitis C (HC) severity. Antibodies (Abs) to microbial glycans could be markers of HC severity caused by the translocation of microbial products. The level of anti-glycan (AG) Abs was analysed in serum samples of patients (*n* = 128) with chronic HC in ELISA using fourteen synthetic glycans present in microbes and adhesins to evaluate the association of Abs with clinical parameters and the efficacy of antiviral treatment. The anti-GlcNAc*β* IgG level was significantly higher in patients with fibrosis (*P* = 0.021) and severe portal inflammation (*P* < 0.001) regardless of other clinical parameters. The ROC curve analysis showed sensitivity of 0.59, specificity of 0.84, and AUC of 0.71 in discriminating F0 from F1–4 (HCV genotype-1b-infected patients). The level of anti-GA2 Abs before Peg-IFN/RBV treatment was significantly higher in nonsustained viral response (non-SVR) to treatment than in SVR (*P* = 0.033). ROC analysis showed sensitivity of 0.62, specificity of 0.70, and AUC of 64. Correlations of AG Abs to clinical parameters were found. The quantification of anti-GlcNAc*β* Abs deserves attention in assessment of the hepatic damage while anti-GA2 Abs may be a sign of immune response related to the antiviral treatment.

## 1. Introduction

Hepatitis C virus (HCV) infection is a global health issue. More than 185 million people worldwide are chronically infected with HCV [1]. The reduction of morbidity and mortality from HC and improving the quality of life of patients with the disease are major challenges in social, economic, and health care programs. The prediction of clinical outcome and selection of an adequate therapy for HC are vital for the management of patients with chronic liver disease. Most HCV infections can evolve into a chronic phase, which may eventually lead to cirrhosis. The modern diagnostics of HC is reliable and is based on the presence of anti-HCV Abs in the sera of patients and the detection of serum HCV RNA (viral load). Viral load is a significant parameter in monitoring the response to antiviral treatment. In the chronic phase of the disease, hepatic fibrosis is developed. Liver biopsy is traditionally considered as a reference standard for the staging of fibrosis. However, this painful method may cause bleeding and, depending on the conditions of obtaining the sample and their performance, may give different results. Noninvasive methods are based on the measurement of liver stiffness by using transient elastography and determination of serum biomarkers. The main drawback of transient elastography in clinical practice is the impossibility of obtaining reliable liver stiffness measurements in around 20% of cases, mainly involving obese patients. Noninvasive approaches such as determination of serum levels of hyaluronic acid, procollagen II N-terminal propeptide, type-IV collagen, and laminine, as well as aspartate aminotransferase/platelet ratio index and FibroTest, are applied in clinical practice for assessment of the severity and monitoring of viral hepatitis. Serum markers exhibit good reproducibility; however, the risk of having false positive results or their variability in the case of concomitant diseases may occur because the markers are HC-nonspecific. Moreover, a single parameter does not provide accurate diagnostics. Thus, combining multiple serum markers and finding new ones deserve research [2]. Since chronic HC patients suffer from other comorbid conditions, including pathological microbial translocation at terminal stages of the disease, the development of new markers for assessing clinical status, association with known parameters, personal monitoring, and treatment is actual.

Hepatotropic noncytopathic HCV is able to persist in infected hosts due to its ability to escape from immune control. The liver damage and disease progression in patients are driven by viral and host factors [3]. The disease progression leads to cirrhosis which is accompanied by the translocation of microbial products and associated complications [4–7]. Microbial translocation is defined as the passage of microorganisms and their products from the gastrointestinal tract to the mesenteric lymph node complex, liver, spleen, and bloodstream because of increased intestinal permeability or damage to the mucosal barrier. Translocation of microbial products promotes the inflammation and damage to the liver because of its anatomical position in the abdomen and vascular system [6]. The liver is populated with a lot of immune cells that are responsible for phagocytosis of bacteria, recognition and presentation of their antigens, production of cytokines, inducing tolerance, and for many other functions. The presence of microbial products such as lipopolysaccharides in the peripheral circulation may promote liver fibrosis *via* different mechanisms [5, 8, 9]. An association between the serum immunoglobulin level and hepatic fibrosis, as well as the treatment outcome in patients with HCV infection, has been reported previously [10, 11]; however, the specificity of Abs remains yet unrevealed.

Glycans are a constituent of lipopolysaccharides, glycoproteins, and glycolipids, and being surface antigens in microorganisms and viruses, they play a key role in the immune recognition of self and foreign antigens. In HCV infection, the humoral immune response to proteins, mostly HCV envelope ones, has been studied, while the response to glycans has remained almost unexplored yet. The investigation of naturally occurring anti-glycan antibodies (AG Abs) is complex because of their heterogeneity and the diversity of natural glycans. The diagnostic potential of AG Abs for hepatocellular carcinoma has been evaluated using printed glycan microarray [12]. Homogenous synthetic polyacrylamide glycoconjugates (PGs) are used in glycobiology and biomedicine to study glycan-binding proteins, including Abs [13].

It is considered that naturally occurring AG Abs in human are induced by enteric microorganisms or their products. The repertoire of human IgG Abs contains specificity not only for microbial antigens but also for a broad spectrum of host glycans that serve as attachment sites for viral and bacterial pathogens and/or exotoxins [14]. Some glycans are cryptic determinants of blood group antigens. Human Abs have been found to bind short fragments of larger glycans. These mono- and oligosaccharides are common in microbial antigens and can be profiled using glycan arrays [15, 16]. The prognostic significance of the level of IgG Abs to some glycans present in enteric bacteria and the relation of Abs to clinical parameters of gastrointestinal cancer patients have been demonstrated by the authors earlier [17]. The present study was launched to find out which of the AG Abs might be noninvasive potential markers of disease progression and response to therapy of chronic HCV-infected patients. The association of the Ab level with clinical parameters of the disease and response to Peg-IFN/RBV therapy were investigated.

## 2. Material and Methods

### 2.1. Patients

The study was carried out in accordance with the ICH GCP Standards and with approval of Tallinn Medical Research Ethics Committee, Estonian Code no. 1130. The written informed consent was obtained from each patient under examination and included the gist of the study, determination of safety/risks, consent for voluntary participation, period, consent to use personal data (name, age, sex, date of birth, and personal identification code), and analyze and publish the obtained results. The anonymous data on persons with the individual code number were examined. The examination and follow-up of patients during the treatment were conducted in accordance with the Estonian national guidelines for the treatment of chronic HC [18]. The diagnosis of HC was based on the presence of anti-HCV Abs in the sera and the detection of serum HCV RNA. One hundred twenty-eight patients with chronic HC were subjected to investigation. Fibrosis stages were histologically verified by the ultrasound-guided liver biopsy. The fibrosis stage and portal inflammation of the liver were assessed according to METAVIR and Ishak scoring systems [19, 20]. The severity of HCV compensated cirrhosis was graded according to the Child-Pugh A classification. None of the patients had been infected with hepatitis A and B viruses and human immunodeficiency virus (HIV) and had no extrahepatic manifestations either. The exclusion criteria for treatment were as follows: age < 18 and >63 years, chronic alcohol abuse, decompensated cirrhosis, current injection of drugs, and depression. All patients were injected with Peg-IFN*α*-2a at a standard weekly dose of 180 *μ*g during 24 weeks, and ribavirin was given per os at a daily dose of 1000 mg or 1200 mg, depending on body weight (below or above 75 kg). Patients were divided into subgroups, according to treatment response: sustained viral response (SVR), nonresponse (NR), and relapse (*R*). SVR was defined as undetectable serum HCV RNA at the end of treatment and at 24 weeks posttreatment. *R* was defined as undetectable HCV RNA at the end of treatment and reappearance within 24 weeks after the end of therapy. NR was defined as detectable HCV RNA during and at the end of treatment (Table 1).

### 2.2. Clinical Analysis of Blood Samples

Clinical parameters were determined according to common guidelines for the health control of chronic HCV-infected patients, observing a standard protocol. Clinical parameters were used for correlations with AG Ab levels. 5 mL venous blood samples was taken from patients during their planned visit to the physician for health control before, during, or after antiviral treatment. Blood serum samples were collected in the Internal Medicine Department of West Tallinn Central Hospital and stored at −60°C in the Virology Department of the National Institute for Health Development. The serum level of HCV RNA (viral load) was determined using the quantitative PCR assay (COBAS® AmpliPrep/COBAS® TaqMan HCV test, Roche). The lower limit of detection was 15 IU/mL. HCV genotypes were determined using the hybridization technique by employing the Versant HCV genotype assay (LiPA) (Bayer HealthCare LLC, Tarrytown, NY). Also, the count/mL of leukocytes, neutrophils, lymphocytes, monocytes, platelets (PLT), and eosinophils and the ratio of neutrophils to lymphocytes were determined and used for correlations with AG Ab levels.

### 2.3. Glycoconjugates

Glycans present in microorganisms or their human adhesins, some of which are expressed in tumors, are listed in Table 2. Fourteen synthetic PGs showing a good reproducibility in assay were applied for the determination of AG Abs. The soluble PGs with Mr 30 kDa were obtained from Lectinity, Russia. Conventional ethanolamide-type PGs of poly[N-(2-hydroxyethyl)acrylamide] (PAA) except TF*β* were used. The TF*β* conjugate contained N-unsubstituted polyacrylamide, TF*β*-pAA, where pAA was an amide-type carrier. TF*β* and GlcNAc*β* conjugates contained 0.1 mol of a saccharide per 1 mol of the carrier. All other PGs contained 0.2 mol of a saccharide per 1 mol of the carrier. Tris(hydroxymethyl)aminomethane-PAA was used as an adequate negative control.

### 2.4. Indirect ELISA

The immunoassay was performed as described earlier [36]. The sera were collected by the centrifugation of clotted venous blood after incubation for 2 h at 37°C. The sera were conserved with sodium azide and kept at 4°C for no longer than three weeks or frozen (−50°C) and thawed once before use. PGs (5 *μ*g/mL) in 0.05 M carbonate buffer, pH 9.2, were applied to the Nunc-Immuno plate (MaxiSorp) and held overnight at 4°C. The plate was incubated with 0.1% bovine serum albumin (BSA) in 0.05 M Tris HCl/0.2 M NaCl/0.02% sodium azide/0.05% Tween 20, pH 7.5 (TBS), for 1 h at 26°C. After washing with TBS, the plate was coated with sera diluted 1 : 25–1 : 100 in TBS/5 mM EDTA/0.25% BSA and incubated for 2 h at 26°C. The dilution 1 : 50 was optimal for selected PGs, showing comparatively low background with control and better validity of results. The plate was washed with TBS, and the goat anti-human IgG-alkaline phosphatase conjugate in TBS was added. The plate was kept for 1.5 h at 26°C and washed. The absorbance (*A*) at 405 nm was measured using a Labsystem Multiskan MCC/340 (Finland) after incubation for 1 h at 26°C with p-nitrophenylphosphate disodium salt (1 mg/mL in 0.1 M glycine buffer, pH 10.3). Each sample was analyzed in duplicate, including analysis with Tris-PAA (negative control). The Ab level was estimated as *A*_test_ minus *A*_control_, where *A*_test_ was the absorbance with the PG and *A*_control_ with Tris-PAA. The variation coefficient was 3%.

### 2.5. Statistical Analysis

Mann-Whitney *U* and chi-squared tests, Spearman's correlation analysis, and binary logistic regression analysis were performed using SPSS (version 22.0). The diagnostic potential of the Ab level was evaluated by the receiver operator characteristic (ROC) curve analysis by using SigmaPlot 12.5. The difference between the groups was considered to be significant when *P* ≤ 0.05.

## 3. Results

**Figure 1:**
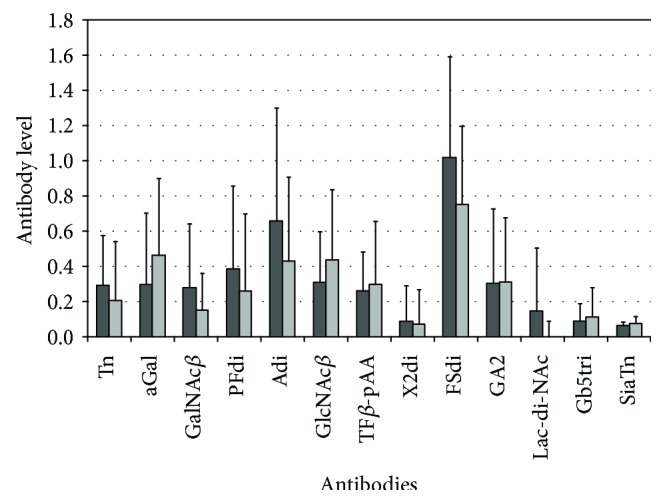
Comparison of anti-glycan IgG levels (mean values) in patients with stages F0 (dark columns) and F1–4 (light columns). The bars indicate the standard deviation (upper SD). The dilution of sera 1 : 50.

**Figure 2:**
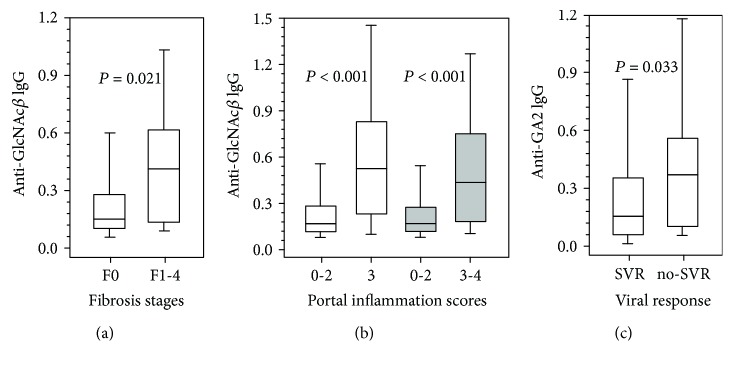
(a) Association of the anti-GlcNAc*β* IgG level with the stages of fibrosis in patients infected with HCV-1b (cut-off 0.334). *Y*-axis: Ab level values (*A*_test_ minus *A*_control_). The lower boundary of the box indicates the 25^th^ percentile data, a line within the box marks the median (*M*), and the upper boundary of the box indicates the 75^th^ percentile. The bars below and above the box indicate the 10^th^ and 90^th^ percentiles, respectively. (b) Association of the anti-GlcNAc*β* IgG level with portal inflammation scores. Light boxes symbolize patients infected with HCV-1b and 3a genotypes in stages F0–2 (cut-off 0.248), and dark boxes denote those in stages F0–4 (cut-off 0.248). (c) Association of the pretreatment level of anti-GA2 IgG with the response to antiviral treatment: SVR—patients with sustained viral response (*M* 0.126); no SVR—patients with nonresponse and relapse (*M* 0.385), cut-off 0.232.

**Figure 3:**
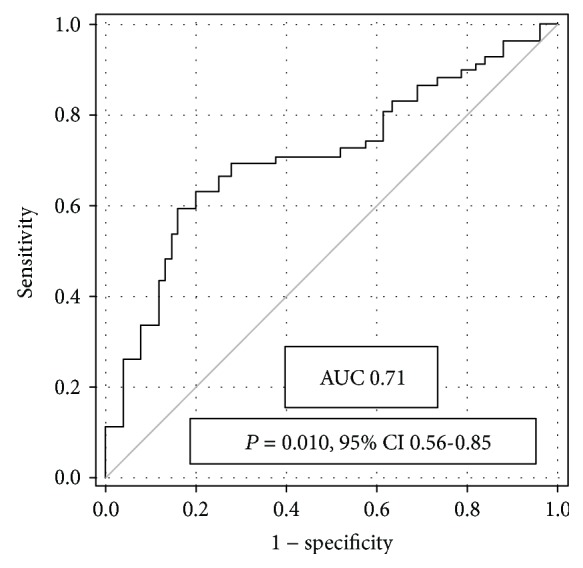
ROC curve analysis of the anti-GlcNAc*β* IgG level in HCV-1b carriers with stages F0 and F1–4.

The AG Ab level values ranged from near zero (anti-TF IgG, not shown) to high for anti-FSdi IgG (Figure 1). The level of Abs was not related to age and gender. A clear association of the level of anti-GlcNAc*β* Abs with fibrosis was established: a significantly higher Ab level was revealed in the serum samples of HCV genotype-1b-infected patients with fibrosis (F1–4) than in patients without fibrosis (F0) (Table 3, Figure 2(a)). In the subgroup infected with HCV-3a, an association of the anti-GlcNAc*β* Ab level with fibrosis was insignificant; however, the Ab level did not depend on the HCV genotype. The level of other AG Abs was not associated with fibrosis stages or exhibited a marginal trend to association. A trend was observed to be higher in fibrosis (F1–4 vs. F0) for the level of anti-*α*Gal (*P* = 0.083) and anti-Gb5tri Abs (*P* = 0.077, F2–4 vs. F0–1). An inverse trend in the latter stages was demonstrated to be lower for anti-PFdi Abs (*P* = 0.055–0.058). An association of the ALP level with the stages of fibrosis in HCV-1b and 3a carriers was observed (Table 3). To evaluate the diagnostic potential of anti-GlcNAc*β* Abs as a marker of the distinction between the patients with fibrosis, the ROC curve analysis was performed (Figure 3). The values of sensitivity and specificity of Abs were comparable with those for ALP (Table 4), but the association of both parameters with fibrosis was independent because no correlation between the levels of anti-GlcNAc*β* Abs and ALP was observed. According to binary logistic regression analysis, the value of odds ratio > 1 indicates correct discrimination of the test (Table 5). In patients with the anti-GlcNAc*β* Ab level ≥ the cut-off value, mean probability in the prediction of fibrosis was equal to 75.9%. In patients with the anti-GlcNAc*β* Ab level < the cut-off value, mean probability in the prediction of the lack of fibrosis was equal to 36.9%.

The portal inflammation of the liver in patients was assessed in scores S0–4, and the level of AG Abs was compared with the results of assessment. It was found that namely anti-GlcNAc*β* Abs were related to the portal inflammation scores regardless of HCV genotype carriers while other AG Abs exhibited no such relation. The level of Abs was significantly higher in scores 3–4 than in scores 0–2. Differences in the Ab level were significant when the scores were compared in patients with earlier stages F0–2 or F0–4 (Table 6, Figure 2(b)).

**Figure 4:**
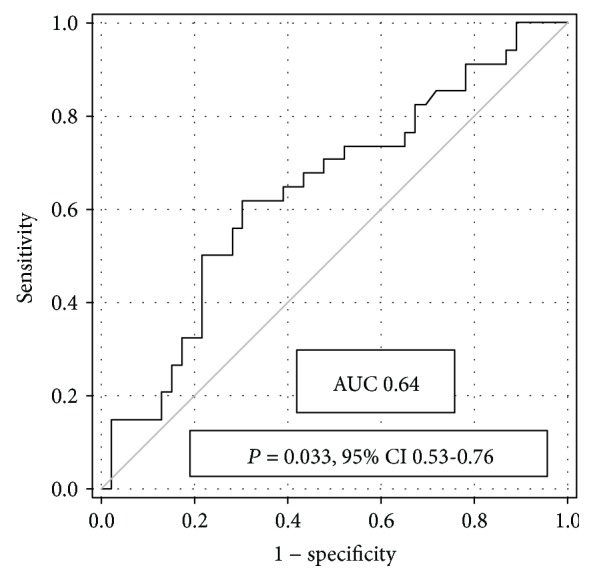
ROC curve analysis of the pretreatment level of anti-GA2 IgG in patients with the response to antiviral treatment: specificity of 0.70; sensitivity of 0.62.

The level of AG Abs was compared with the response of patients to Peg-IFN/RBV treatment. It was found that the pretreatment anti-GA2 IgG level was significantly higher in patients with NR and *R* in combination (non-SVR) than in patients with SVR (*P* = 0.033, Figure 2(c)). The pretreatment level of anti-GA2 IgG in patients with HCV-3a did not differ significantly from that in patients with HCV-1b and was not associated with the fibrosis stage either. The pretreatment level of other AG Abs was not associated with the response of patients to Peg-IFN/RBV treatment. In the discrimination of SVR vs. non-SVR, the ROC curve analysis showed values of specificity, sensitivity, and AUC present in Figure 4. In binary logistic regression analysis of the anti-GA2 Ab level, the value of odds ratio was >1 (Table 5). Mean probability in the prediction of SVR in Peg-IFN/RBV treatment of patients having the anti-GA2 Ab level < the cut-off value was equal to 36.0% while in the prediction of non-SVR among patients with the level ≥ the cut-off value, the probability was equal to 50.8%.

**Figure 5:**
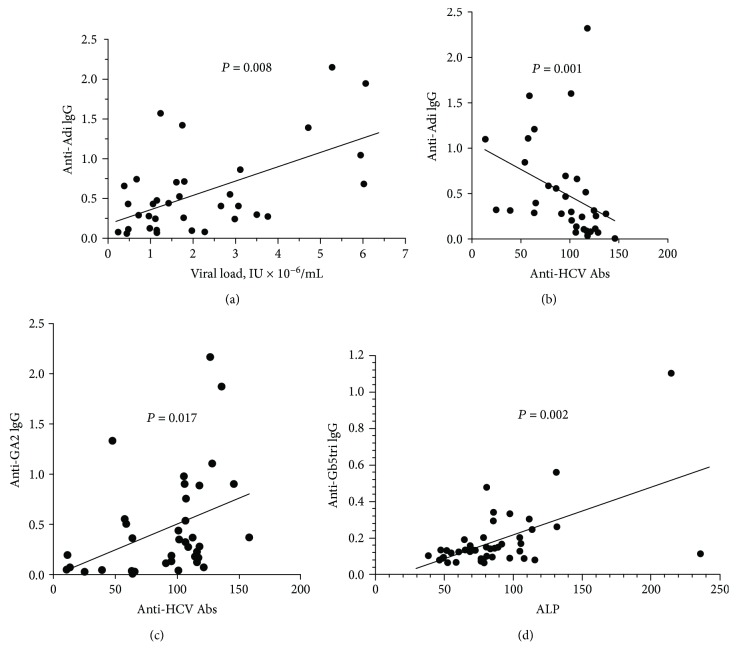
Correlation between the anti-AG Ab level and clinical parameters. (a) The anti-Adi Ab level vs. viral load in HCV-1b carriers in stages F0–1. (b) The anti-Adi Ab level vs. the anti-HCV Ab level. (c) The anti-GA2 Ab level vs. the anti-HCV Ab level. (d) The anti-Gb5tri Ab level vs. the ALP level in stages F1–4.

The AG Ab level data were analysed for correlation with clinical parameters which are usually used for the health control of chronic HC patients. The correlation between the anti-Adi IgG level and viral load was detected in HCV-1b carriers but not in HCV-3a carriers. The correlation was observed in patients with stages F0–4 and in the earlier stages of the disease F0 and F0–1. Besides, the anti-Adi IgG level correlated inversely with the level of anti-HCV Abs, and this agreed with the inverse correlation between viral load and anti-HCV Abs (Table 7, Figures 5(a) and 5(b)). It was found that the anti-GA2 IgG level correlated with the levels of anti-HCV Abs and AFP. The level of anti-Gb5tri and anti-TF*β* Abs correlated with that of ALP in patients with fibrosis (Figures 5(c) and 5(d), Table 7). Besides, correlations of the level of anti-Adi Abs vs. the count of monocytes, anti-Tn Abs vs. the count of lymphocytes, and anti-GalNAc*β* Abs vs. the bilirubin level were observed.

The influence of Peg-IFN/RBV treatment on the level of AG Abs was investigated in dynamics of HCV-1b-infected patients (*n* = 56). The treatment caused both the increase and decrease of the Ab level or had no effect. The level of AG Abs in the serum of some patients had increased after treatment two- to threefold. In some patients, the increased level of AG Abs at the beginning of treatment had dropped to the initial values after the end of treatment. The increase or decrease of the Ab level ≥ 50% after the end of treatment, compared with their pretreatment level, was used as a criterion of assessment of Ab level change. The share of patients whose treatment elevated the level of AG Abs (except anti-Adi IgG) was higher in stages F0–1 (*n* = 31) than in F2–4 (*n* = 25) (Table 8); however, a significant difference was marked only for anti-GA2 Abs (chi-squared test, *P* = 0.048). Also, the posttreatment level of anti-GA2 Abs was mostly increased in the SVR subgroup (*n* = 26) unlike the non-SVR subgroup (*n* = 30, *P* = 0.036). Stage-matched research with more number of patients will be required to draw final conclusions.

## 4. Discussion

### 4.1. Association of the AG Ab Level with Fibrosis and Inflammation

Naturally occurring Abs against various glycans are constantly stimulated by gut microbiota in humans. In immune response under pathological conditions, Abs to microbial glycans are preferable markers in the diagnostics and monitoring of the disease as the level of circulating glycans may be too low to be detected or glycans are hidden in immune complexes. The total serum IgG level is a surrogate marker of disease severity and treatment outcome in patients with chronic HC. The association of the serum IgG level with hepatic fibrosis in HCV-infected patients has been established [10, 11]; however, the specificity of Abs remains yet unclear. Based on literature data and our earlier publications, we selected fourteen glycans that are listed in Table 2. The main criteria of selection were the bacterial expression of glycans, targeting for human Abs, and cellular receptors (adhesins), as well as a possible involvement in autoimmune diseases (Abs to cellular receptors or human-mimic antigens) [14]. We investigated earlier the specificity and cross-reactivity of IgG antibodies to related and nonrelated glycans, including PG affinity-isolated Abs from the sera of patients with gastrointestinal cancer (Table 2). In the present study, synthetic PGs containing natural determinants were applied for determination of the level of specific AG Abs. Synthetic PG models are homogenous antigens with a single reiterative glycotope; therefore, they have certain advantages over natural antigens containing usually different determinants (good reproducibility of assay, low background, and stability). A significantly higher level of anti-GlcNAc*β* IgG was observed in fibrosis in patients infected with HCV-1b genotype. The diagnostic potential of Abs in discriminating patients in stage F0 from those in stages F1–4 was evaluated using the ROC curve and binary logistic regression analyses yielding parameters shown in Tables 4 and 5. The likelihood in the prediction of fibrosis was high, i.e., in patients with the high anti-GlcNAc*β* Ab level, mean probability in the prediction of fibrosis was equal to 75.9%. In the group of patients with the low anti-GlcNAc*β* Ab level, the outcome was less likely; mean probability in the prediction of the lack of fibrosis was equal to 36.9%.

There were significant differences in the level of anti-GlcNAc*β* Abs between patients with portal inflammation as well (Table 6). The increased anti-GlcNAc*β* IgG level was found to be associated with portal inflammation scores (S3–4 *vs.* S0–2) which were assessed in disease stages F0–2 and F0–4. The anti-GlcNAc*β* IgG level did not correlate with the other clinical parameters, including neutrophil/lymphocyte ratio, which reflects a common inflammatory response. The neutrophil/lymphocyte ratio was not associated with the portal inflammation score either. Thus, the high level of anti-GlcNAc*β* Abs was associated with fibrosis pathogenesis and portal inflammation regardless of clinical parameters examined in the present study. This indicated the complicity of Ab response to GlcNAc*β* in hepatic damage. However, in the progression of other viral infections (HBV) and nonviral hepatic diseases (nonalcoholic fatty liver disease, nonalcoholic steatohepatitis, and chronic alcohol abuse), which lead to liver damage, increased intestinal permeability, bacterial overgrowth, and microbial translocation, the anti-GlcNAc*β* Ab level might also exceed normal values.

GlcNAc*β* is a dominant epitope of the group A streptococcal antigen that stimulates pathological cross-reactive autoantibodies [48]. The diagnostic and prognostic potential of anti-GlcNAc*β* Abs in ELISA with GlcNAc*β*-PG (the glycoconjugate used in the present study) has been demonstrated for rheumatic fever and the rheumatic heart disease of *Streptococcus* A carriers [49]. In the present study, neither streptococcal infections nor the rheumatic heart disease and other rheumatic manifestations were registered in HCV-infected patients whose anti-GlcNAc*β* IgG level exceeded median values. However, *Streptococcus* A-mediated infections including their rheumatic manifestations should be taken into consideration as they may influence the level of Abs. The prevalence of potentially pathogenic Streptococcaceae bacteria with the reduction of beneficial bacteria in the fecal samples of patients with cirrhosis has been demonstrated earlier. Moreover, a positive correlation between Child-Turcotte-Pugh score and the share of Streptococcaceae in gut microbiota has been observed [50]. We suggest that the translocation of bacterial GlcNAc*β*-bearing antigens through the intestinal barrier in fibrosis can stimulate Ab production and contribute to further liver damage, causing chronic inflammatory responses.

The functionality of antibacterial Abs in the progression of HC has been shown to worsen, and bacterial products may have contributed to the development of liver fibrosis [9]. The functionality of anti-*α*Gal Abs in the pathogenesis of different diseases, including HC, has been studied more thoroughly before [9, 39, 40]. Anti-Gal Abs may play an important role in the survival of Gram-negative enterobacteria by blocking their lysis *via* an alternative complement pathway [51]. The reduced ability of *α*Gal-specific Abs to complement-mediated lysis of *α*Gal-bearing bacteria in patients with liver cirrhosis has been associated with the abnormal glycosylation of Abs [9, 52]. It is known that natural Abs to GlcNAc*β*1-6 polysaccharide (a broadly distributed antigen among human pathogens) are common in humans but these Abs do not protect against infection due to the lack of deposition of complement opsonins [43]. As in case of anti-*α*Gal Abs, the overproduction of fibrosis-related anti-GlcNAc*β* IgGs may be due to the disability of Abs to neutralize GlcNAc*β* glycan-bearing bacteria as a consequence of abnormal antibody glycosylation. Analysis of antibody glycosylation by using lectins could confirm this supposition. The mean value of the anti-*α*Gal IgG level in patients with fibrosis was increased (Figure 1), but we observed only a weak trend to the higher level of anti-*α*Gal Abs in fibrosis. This is not in contradiction with literature results [52]. The *α*Gal glycan belongs to blood group-related glycans. Anti-*α*Gal Abs are heterogenic, and their reactivity depends on the blood group phenotype [53]. Heterogenic and polyreactive Abs may not be as suitable for diagnostic purposes as oligoreactive Abs and viral hepatitis-related oligoclonal Abs [54].

We showed that there was a low or close-to-background level of anti-TF IgG in the sera of most patients under study in using TF-PG that binds with the asialoglycophorin-specific monoclonal Abs. Negative results of IgG binding to TF were also demonstrated for most commercial intravenous and subcutaneous immunoglobulin preparations as well as for the IgG Abs pool purified from donors' sera [14, 16].

### 4.2. Association of the AG Ab Level with Other Clinical Parameters

There were statistically significant correlations between the level of some AG Abs and clinical parameters. We examined the scientific literature to discuss briefly the obtained results. The unusual correlation between the anti-Adi IgG level and viral load was revealed. In addition, the anti-Adi IgG level inversely correlated with the level of anti-HCV Abs. The glycan structures in HCV glycoproteins have been poorly studied yet. It is known that the glycosylation patterns of viral glycoproteins depend on host cells and affect IgG binding [55]. The enveloped viruses can incorporate the ABO histoblood group antigens of the host cells [56]. This might stimulate the antibody response to blood group-related xenogeneic glycans. Interestingly, the adaptive antibody response to Adi and FSdi glycans has been demonstrated in cancer patients after immunization with pox virus vaccine [57].

The level of anti-Gb5tri IgG in the serum of patients with hepatocellular carcinoma was shown to be significantly higher than in healthy individuals (the glycan microarray assessment, glycan no. 42) [12]. However, the authors did not consider these Ab potential biomarkers. We observed a trend to be higher in the latter stages of fibrosis for anti-Gb5tri IgG. Gb5tri and TF*β* are structurally related glycans having the same terminal disaccharide (Table 2); therefore, the Ab cross-reactivity to both glycans was demonstrated earlier [25] and confirmed by correlation between the Ab level in the present study (*r* = 0.57, *P* = 0.008, *n* = 69). In the present study, the correlation between the level of ALP on the one hand and the levels of anti-Gb5tri and anti-TF*β* Abs on the other hand was observed (Table 7). It is possible that this correlation is only indirect if ALP and Abs do not have an interconnection but are closely dependent on or correlate with another unknown pathophysiological parameter. Over the past few years, the role of intestinal ALP in maintaining gut homeostasis has been established [58]. ALP has a more general anti-inflammatory role as it is capable of dephosphorylating potentially deleterious nucleotide phosphates and endotoxin lipopolysaccharide. The immune complexes of ALP with IgG have been a subject of research in different pathologies, including bacterial infections [59]. According to our observations, the level of ALP is not associated with portal inflammation score. It is known that the serum level of ALP depends on different pathophysiological parameters that may influence its diagnostic potential. Nevertheless, statistical analysis showed that there were significant fibrosis-associated differences in the ALP level at values of sensitivity and specificity in the range of 0.64–0.77 (Tables 3 and 4). This may be due to the fact that the group of patients under study was relatively homogeneous. Thus, the observed correlations between the AG Ab level and clinical parameters may reflect their indirect relationship or may be indicative of unknown structural-functional interrelations. All these observations may serve as a basis for various assumptions and need further research, which goes beyond the scope of the current study.

### 4.3. Relation of the Pretreatment AG Ab Level to Response to Peg-IFN/RBV Treatment and the Influence of Treatment on the AG Ab Level

The levels of serum gamma globulin and total IgG were found to significantly decrease in complete responders to IFN treatment [11]. We indicated an association of specific anti-GA2 IgG with the antiviral response. The level of Abs before treatment was significantly lower in the SVR subgroup than in the non-SVR subgroup (Figure 2(c)). The correlation between the level of anti-GA2 and anti-HCV Abs was demonstrated (Figure 5(c), Table 7) while an inverse correlation of the anti-GA2 level with the viral load was insignificant. The association of the anti-GA2 Ab level with response to antiviral treatment remains yet unclear. The terminal disaccharide of GA2 (GalNAc*β*1-4Gal*β*) can serve as a receptor of pathogens and their products [14, 28]. The anti-GA2 IgGs might belong to autoantibodies against the altered self GM2 ganglioside as a consequence of its desialylation by exogenous neuraminidases.

Anti-GA2 Abs could be a potential marker of the antiviral treatment efficacy as shown in the ROC curve and binary logistic regression analyses (Figure 4, Table 5). Mean probability in the prediction of SVR in Peg-IFN/RBV treatment among patients with the low anti-GA2 Ab level was low (36.0%), while in the prediction of non-SVR in patients having a high anti-GA2 Ab level, probability was higher (50.8%). These results require additional study on a larger contingent of patients for a separate comparison of the subgroups NR and *R* vs. SVR.

Type I interferons (IFN*α* and IFN*β*) are effector cytokines that modulate innate and adaptive immune responses. The disparate effects of type I IFNs on bacterial infections are in marked contrast to their well-established protective roles in most viral infections [60]. The IFN*α* treatment may be accompanied by a variety of side effects which may make it harder to maintain the dose needed to achieve the desirable therapeutic effect, and sometimes, side effects can outweigh clinical benefit from treatment [61]. For example, the elevation of anti-asialo-GM1 (GA1) and anti-GM1 Ab levels in the serum and induction of peripheral neuropathy during Peg-IFN*α* treatment of the patient with HC have been reported [62]. According to our observations, the increase of the AG Ab level occurred in 8–50% of patients, the decrease in 0–33%, and weak or no change was marked in 45–79% of patients (Table 8). The increase of the AG Ab level after Peg-IFN/RBV treatment was noticed more frequently in earlier stages F0–1, and this was significant for anti-GA2 Abs. Besides, the increased posttreatment level of anti-GA2 Abs prevailed in the SVR subgroup compared with no-SVR. The profile of change was complex that may be due to the individual responsiveness and ambivalent effect of type I IFNs on the regulation of immune homeostasis [60]. To elucidate the relation of AG Abs to HC exacerbations, patients will be subjected to a long-term follow-up during further research.

## 5. Conclusions

In the present study, two of the fourteen AG Abs investigated, namely anti-GlcNAc*β* and anti-GA2 Abs, were selected as potential markers of liver damage and Peg-IFN/RBV antiviral therapy, respectively. Association of the level of other AG Abs in relation to hepatic damage and antiviral treatment was not revealed. It was established that there were significant differences in the level of anti-GlcNAc*β* IgG between (1) patients with fibrosis and those without fibrosis and (2) patients with severe portal inflammation (scores S3–4) and those having scores S0–2 examined in stages F0–2 or F0–4. The diagnostic potential of Abs in discriminating patients with fibrosis from those without fibrosis was evaluated using the ROC curve and binary logistic regression analyses. The likelihood in the prediction of fibrosis was high in patients with the high anti-GlcNAc*β* Ab level. The anti-GA2 Ab level may be a potential marker of a response to treatment as shown by using both analyses. The immunoassay using GlcNAc*β*-PG showed a good reproducibility, and in combination with other routine clinical parameters, it may be a useful supplement for the assessment of hepatic damage. The level of anti-GlcNAc*β* Abs in chronic HC should be investigated in a long-term follow-up particularly in cases when the disease progression may lead to cirrhosis and mediated bacterial infections [4], as well as in screening for antibacterial treatment. The level of anti-GA2 Abs before treatment and its dynamics may be one of the signs of immune response related to the antiviral treatment efficacy. The level of anti-Adi Abs correlated with the viral load, back correlated with the level of anti-HCV Abs, and may indicate the involvement of Abs in HC pathogenesis.

## Figures and Tables

**Table 1:** Characteristics of the patients and their clinical parameters (mean ± SD).

Patients, mean age ± SD: 41.1 ± 11.3	*n*
Males	77
Females	51
HCV genotypes	
1b	82
3a	46
Fibrosis stages	
F0	56
F1	26
F2	19
F3	7
F4	20
Portal inflammation	
Scores S3–4 (moderate and marked inflammation in all portal areas)	51
Scores S0–2 (without inflammation or with mild and moderate inflammation in some or all portal areas)	68
Response to Peg-IFN/RBV treatment	
SVR	69
NR	18
*R*	22
Clinical parameters	Mean ± SD
Viral load, IU × 10^6^/mL	1.81 ± 2.01
Anti-HCV Abs, S/CO	93.5 ± 37.8
Aspartate aminotransferase (AST), IU/L	71.4 ± 77.1
Alanine aminotransferase (ALT), IU/L	103.1 ± 93.3
AST/ALT ratio	0.81 ± 0.78
AST/PLT ratio	0.37 ± 0.43
Bilirubin, *μ*mol/L	12.5 ± 5.9
Gamma-glutamyltransferase, IU/L	73.4 ± 78.9
Alkaline phosphatase (ALP), IU/L	80.0 ± 32.4
Ferritin, *μ*g/L	155.5 ± 138.3
Thyroid-stimulating hormone, mIU/L	1.41 ± 0.86
Alpha-fetoprotein (AFP), *μ*g/L	2.89 ± 1.81

**Table 2:** Brief characterization of glycans and reactivity of human AG IgGs.^∗^

Abbreviations, nomenclature	Origin and reactivity of human IgG Abs
TF, TF*α*, Gal*β*1-3GalNAc*α*	Core 1, mucin-type glycan, expressed in tumors. TF is rarely expressed in gut bacteria and was found in *Bacteroides ovatus* and *E. coli*. Is present in streptococcal polysaccharides and HCV glycoproteins. In most cases, no or weak reactivity of intravenous IgG Abs to TF was observed [14, 21–24].
TF*β*, Gal*β*1-3GalNAc*β*	A constituent of human glycolipids (GM1, Gb5). Present in *Bacteroides ovatus* and *E. coli*. Cross-reactivity of Ab to TF*αβ*, GA1, and Gb5tri was shown [20, 25].
Gb5tri, Gal*β*1-3GalNAc*β*1-3Gal	The terminal trisaccharide of Gb5 is expressed in human embryonic and tumor cells and erythrocytes [26, 27].
GA2, GalNAc*β*1-4Gal*β*1-4Glc*β*	Receptor for pathogenic bacteria [28]. Insignificant cross-reactivity to related glycans was observed for serum IgG [29].
Lac-di-NAc, GalNAc*β*1-4GlcNAc*β*	Glycans are expressed in tumor cells and parasites [30, 31]. Adhesin for *Helicobacter pylori* [32].
Tn, GalNAc*α*	Mucin-type glycan is related to different diseases and is expressed in tumor cells. Is present in different microorganisms and is a target for mucin-degrading bacteria [21, 33, 34]. Monoreactivity to Tn or partial cross-reactivity to related glycans was shown for anti-Tn IgG [35, 36].
SiaTn, Neu5Ac*α*2-6GalNAc*α*	Tumor-associated glycan. An adhesin for viruses and mucin-degrading bacteria [34]. Cross-reactivity to Tn was shown for anti-SiaTn IgG [35, 37].
Adi, GalNAc*α*1-3Gal*β*	A constituent of the A blood group antigen and is a ligand for the spike protein of rotavirus [38]. Is presumably xenogeneic glycan for human. Monoreactivity to Adi or cross-reactivity to related glycans was revealed for anti-Adi IgG [36].
*α*Gal, Bdi, Gal*α*1-3Gal*β*	A constituent of the B blood group antigen. Xenogeneic for human and is related to autoimmune disorders. *α*Gal is expressed in pathogenic bacteria (*E. coli*, *Klebsiella*, and *Salmonella*) [39]. Cross-reactivity to related glycans was shown for anti-*α*Gal IgG [36, 40, 41].
FSdi (terminal part of Forssman glycolipid), GalNAc*α*1-3GalNAc*β*	Xenogeneic for human is considered, but recently Fs was revealed in erythrocytes of A(pae) subgroup [42]. Fs-related cross-reactive antigens are present in microbes and animals. Cross-reactivity to related glycans was shown for serum IgG [36].
GlcNAc*β*	A determinant of *β*-poly-N-acetyl-glucosamine of pathogenic microbes [43]. Is present in human mucin-core structures. Enteric bacteria degrade mucins and produce GlcNAc*β* glycans [34, 44]. Partial IgG cross-reactivity to GalNAc*β* was revealed [29].
GalNAc*β*	GalNAc*β* glycans are synthesized by enteric bacteria [44]. Cross-reactivity to X2_di_ was observed for anti-GalNAc*β* IgG [29].
PFdi (outer part of Para-Forssman glycolipid), GalNAc*β*1-3GalNAc*β*	PF is a minor component of human erythrocytes [45]. Is a constituent of the cyst wall antigen of *Giardia intestinalis* [46]. Monoreactivity to PFdi was shown for IgG Abs [29].
X2_di_ (terminal part of X2 glycolipid), GalNAc*β*1-3Gal*β*	X2 is a minor component of human erythrocytes and other cells. Is associated with the P blood group phenotype [47]. Streptococcal receptor polysaccharides contain similar structures [23]. Monoreactivity to X2_di_ was shown for IgG Abs [29].

^∗^In general, the IgG immune profile reflects the high immunogenicity of terminal Gal, GalNAc, and GlcNAc moieties. Besides, IgGs contain specificity to human glycans that are adhesins for viral and bacterial pathogens and/or exotoxins [14].

**Table 3:** Association of the level of anti-GlcNAc*β* Abs and ALP with the stages of fibrosis.

Parameter	Genotype	Stage (*F*)	Median (*M*)	*P*
Anti-GlcNAc*β*	1b	0	0.155	
		1–3	0.377	0.055 (F0 *vs.* F1–3)
		1–4	0.389	0.021 (F0 *vs.* F1–4)

ALP	1b	0	67	
		1–4	80	0.025 (F0 vs. F1–4)
		0–1	67	
		2–4	86	0.002 (F0–1 vs. F2–4)
	1b and 3a	0	67	
		1–4	81	0.008 (F0 vs. F1–4)
		0–1	68	
		2–4	86	0.001 (F0–1 vs. F2–4)

**Table 4:** Diagnostic parameters of patients in stage F0 vs. F1–4.

Parameter	Genotype	Cut-off	Sensitivity	95% CI	Specificity	95% CI
Anti-GlcNAc*β*	1b	0.334	0.59	0.39–0.78	0.84	0.64–0.95

ALP	1b	74.5	0.64	0.41–0.83	0.76	0.50–0.93
	1b and 3a	72.5	0.64	0.45–0.80	0.77	0.56–0.91

**Table 5:** Binary logistic regression analysis of the Ab level in the discrimination of patients with fibrosis and predicting treatment response.

Abs (variables)	Cut-off	Characteristics	Odds ratio (Exp B)	95% CI	*P*
Anti-GlcNAc*β*	0.334	F0, F1–4	4.11	1.49–23.51	0.024
Anti-GA2	0.232	SVR, non-SVR	4.26	1.12–16.15	0.033

**Table 6:** Association of the level of anti-GlcNAc*β* Abs with portal inflammation score.

Abs	Genotype	Stage (*F*)	Score (*S*)	Median (*M*)	*P*
GlcNAc*β*	1b	0–4	0–2	0.144	0.004 (S0–2 *vs.* S3–4)
			3–4	0.513	
	3a	0–4	0–2	0.212	0.002 (S0–2 *vs.* S3–4)
			3–4	0.487	
	1b and 3a	0–2	0–2	0.166	<0.001 (S0–2 *vs.* S3)
			3	0.531	
		0–4	0–2	0.166	<0.001 (S0–2 *vs.* S3–4)
			3–4	0.474	

**Table 7:** Correlation between the AG Ab level and clinical parameters of patients.

Abs	Parameter	*n*	*r*_s_^∗^	*P*
Adi in F0	Viral load	25	0.47	0.017
Adi in F0–1	Viral load	35	0.44	0.008
Adi in F0–4	Viral load	62	0.37	0.012
Adi in F0–4	Anti-HCV Abs	35	−0.61	0.001
Adi	Monocytes (count/mL)	54	−0.32	0.017
GA2 in F0–4	Anti-HCV Abs	38	0.39	0.017
GA2 in F0–4	AFP	29	0.63	<0.001
Gb5tri in F0–4	ALP	67	0.37	0.002
Gb5tri in F1–4	ALP	43	0.46	0.002
TF*β* in F1–4	ALP	43	0.38	0.025
Viral load	Anti-HCV Abs	38	−0.40	0.042
Tn	Lymphocytes (count/mL)	35	−0.52	0.002
GalNAc*β*	Bilirubin	53	0.31	0.024

^∗^ *r*_s_ denotes Spearman's correlation coefficient.

**Table 8:** Share of patients exhibiting the AG Ab level change after Peg-IFN/RBV therapy.

Ab level in patients, %	Adi	GlcNAc*β*	GA2	Bdi	GalNAc*β*	PFdi	Gb5tri
Increased	45	36	30	46	21	50	8
Decreased	5	9	13	8	0	5	33
Weak or no effect	50	55	57	46	79	45	59
Increased in F0–1	45	42	47	57	25	58	20
Increased in F2–4	44	27	8	33	17	40	3

## Data Availability

Data can be submitted by corresponding author in case of a request. General characteristics of the patients and their clinical parameters are presented in Table 1.

## Abbreviations

*A*: Absorbance
AFP: Alpha-fetoprotein
AG Abs: Anti-glycan antibodies
ALP: Alkaline phosphatase
HCV: Hepatitis C virus
*M*: Median
NR: Nonresponse
Peg-IFN/RBV: Pegylated interferon-*α*/ribavirin
PGs: Polyacrylamide glycoconjugates
PLT: Platelets
*R*: Relapse
ROC: Receiver operator characteristic
SD: Standard deviation
SVR: Sustained viral response.

## References

[1] Mohd Hanafiah K., Groeger J., Flaxman A. D., Wiersma S. T. (2013). Global epidemiology of hepatitis C virus infection: new estimates of age-specific antibody to HCV seroprevalence. *Hepatology*.

[2] Li C., Li R., Zhang W. (2018). Progress in non-invasive detection of liver fibrosis. *Cancer Biology & Medicine*.

[3] Larrubia J. R., Moreno-Cubero E., Lokhande M. U. (2014). Adaptive immune response during hepatitis C virus infection. *World Journal of Gastroenterology*.

[4] Papp M., Norman G. L., Vitalis Z. (2010). Presence of anti-microbial antibodies in liver cirrhosis--a tell-tale sign of compromised immunity?. *PLoS One*.

[5] Sandler N. G., Koh C., Roque A. (2011). Host response to translocated microbial products predicts outcomes of patients with HBV or HCV infection. *Gastroenterology*.

[6] Seo Y. S., Shah V. H. (2012). The role of gut-liver axis in the pathogenesis of liver cirrhosis and portal hypertension. *Clinical and Molecular Hepatology*.

[7] Wiest R., Lawson M., Geuking M. (2014). Pathological bacterial translocation in liver cirrhosis. *Journal of Hepatology*.

[8] D'Ettorre G., Douek D., Paiardini M., Ceccarelli G., Vullo V. (2012). Microbial translocation and infectious diseases: what is the link?. *International Journal of Microbiology*.

[9] Lamontagne A., Long R. E., Comunale M. A. (2013). Altered functionality of anti-bacterial antibodies in patients with chronic hepatitis C virus infection. *PLoS One*.

[10] Watt K., Uhanova J., Gong Y. (2004). Serum immunoglobulins predict the extent of hepatic fibrosis in patients with chronic hepatitis C virus infection. *Journal of Viral Hepatitis*.

[11] Maruyama S., Hirayama C., Horie Y. (2007). Serum immunoglobulins in patients with chronic hepatitis C: a surrogate marker of disease severity and treatment outcome. *Hepato-Gastroenterology*.

[12] Wu C. S., Yen C. J., Chou R. H. (2012). Cancer-associated carbohydrate antigens as potential biomarkers for hepatocellular carcinoma. *PLoS One*.

[13] Bovin N. V. (1998). Polyacrylamide-based glycoconjugates as tools in glycobiology. *Glycoconjugate Journal*.

[14] Schneider C., Smith D. F., Cummings R. D. (2015). The human IgG anti-carbohydrate repertoire exhibits a universal architecture and contains specificity for microbial attachment sites. *Science Translational Medicine*.

[15] Bovin N., Obukhova P., Shilova N. (2012). Repertoire of human natural anti-glycan immunoglobulins. Do we have auto-antibodies?. *Biochimica et Biophysica Acta*.

[16] Dotan N., Altstock R. T., Schwarz M., Dukler A. (2006). Anti-glycan antibodies as biomarkers for diagnosis and prognosis. *Lupus*.

[17] Smorodin E., Sergeyev B., Klaamas K., Chuzmarov V., Kurtenkov O. (2013). The relation of the level of serum anti-TF, -Tn and -alpha-Gal IgG to survival in gastrointestinal cancer patients. *International Journal of Medical Sciences*.

[18] Margus B., Salupere R., Ott K. (2007). Kroonilise C-hepatiidi ravijuhend. *Eesti Arst*.

[19] Bedossa P., Poynard T. (1996). An algorithm for the grading of activity in chronic hepatitis C. The METAVIR Cooperative Study Group. *Hepatology*.

[20] Goodman Z. D. (2007). Grading and staging systems for inflammation and fibrosis in chronic liver diseases. *Journal of Hepatology*.

[21] Springer G. F., Desai P. R., Ghazizadeh M., Tegtmeyer H. (1995). T/Tn pancarcinoma autoantigens: fundamental, diagnostic, and prognostic aspects. *Cancer Detection and Prevention*.

[22] Henderson G., Ulsemer P., Schöber U. (2011). Occurrence of the human tumor-specific antigen structure Gal*β*1-3GalNAc*α*- (Thomsen–Friedenreich) and related structures on gut bacteria: prevalence, immunochemical analysis and structural confirmation. *Glycobiology*.

[23] Yoshida Y., Palmer R. J., Yang J., Kolenbrander P. E., Cisar J. O. (2006). Streptococcal receptor polysaccharides: recognition molecules for oral biofilm formation. *BMC Oral Health*.

[24] Brautigam J., Scheidig A. J., Egge-Jacobsen W. (2013). Mass spectrometric analysis of hepatitis C viral envelope protein E2 reveals extended microheterogeneity of mucin-type O-linked glycosylation. *Glycobiology*.

[25] Smorodin E. P., Kurtenkov O. A., Sergeyev B. L., Klaamas K. V. (2011). The characterization of cross-reactive antibodies to Thomsen-Friedenreich *α*/*β* and related glycan-conjugates with polyacrylamide carriers in patients with gastrointestinal cancer. *Journal of Clinical & Cellular Immunology*.

[26] Chang W. W., Lee C. H., Lee P. (2008). Expression of Globo H and SSEA3 in breast cancer stem cells and the involvement of fucosyl transferases 1 and 2 in Globo H synthesis. *Proceedings of the National Academy of Sciences of the United States of America*.

[27] Wright A. J., Andrews P. W. (2009). Surface marker antigens in the characterization of human embryonic stem cells. *Stem Cell Research*.

[28] Thomas R. J. (2010). Receptor mimicry as novel therapeutic treatment for biothreat agents. *Bioengineered Bugs*.

[29] Smorodin E. P., Sergeyev B. L., Kurtenkov O. A. (2014). The characterization of IgG antibodies to GalNAc beta-terminated glycans of gastric cancer survivors. *Experimental Oncology*.

[30] Hirano K., Matsuda A., Shirai T., Furukawa K. (2014). Expression of LacdiNAc groups on N-glycans among human tumors is complex. *BioMed Research International*.

[31] van den Berg T. K., Honing H., Franke N. (2004). LacdiNAc-glycans constitute a parasite pattern for galectin-3-mediated immune recognition. *The Journal of Immunology*.

[32] Rossez Y., Gosset P., Boneca I. G. (2014). The LacdiNAc-specific adhesin laba mediates adhesion of *Helicobacter pylori* to human gastric mucosa. *The Journal of Infectious Diseases*.

[33] Ju T., Otto V. I., Cummings R. D. (2011). The Tn antigen-structural simplicity and biological complexity. *Angewandte Chemie*.

[34] Tailford L. E., Crost E. H., Kavanaugh D., Juge N. (2015). Mucin glycan foraging in the human gut microbiome. *Frontiers in Genetics*.

[35] Smorodin E. P., Kurtenkov O. A., Sergeyev B. L., Pazynina G. V., Bovin N. V. (2004). Specificity of human anti-carbohydrate IgG antibodies as probed with polyacrylamide-based glycoconjugates. *Glycoconjugate Journal*.

[36] Smorodin E. P., Kurtenkov O. A., Sergeyev B. L., Branovets J. S., Izotova J. G., Formanovsky A. A. (2011). Specificity of serum anti-A (di) IgG antibodies from patients with gastrointestinal cancer. *Journal of Immunoassay & Immunochemistry*.

[37] Julien S., Videira P. A., Delannoy P. (2012). Sialyl-tn in cancer: (how) did we miss the target?. *Biomolecules*.

[38] Liu Y., Huang P., Tan M. (2012). Rotavirus VP8∗: phylogeny, host range, and interaction with histo-blood group antigens. *Journal of Virology*.

[39] Galili U., Mandrell R. E., Hamadeh R. M., Shohet S. B., Griffiss J. M. (1988). Interaction between human natural anti-alpha-galactosyl immunoglobulin G and bacteria of the human flora. *Infection and Immunity*.

[40] Galili U. (2013). Anti-Gal: an abundant human natural antibody of multiple pathogeneses and clinical benefits. *Immunology*.

[41] Smorodin E. P., Kurtenkov O. A., Shevchuk I. N., Tanner R. H. (2005). The isolation and characterization of human natural *α*Gal‐specific IgG antibodies applicable to the detection of *α*Gal‐glycosphingolipids. *Journal of Immunoassay & Immunochemistry*.

[42] Svensson L., Hult A. K., Stamps R. (2013). Forssman expression on human erythrocytes: biochemical and genetic evidence of a new histo-blood group system. *Blood*.

[43] Cywes-Bentley C., Skurnik D., Zaidi T. (2013). Antibody to a conserved antigenic target is protective against diverse prokaryotic and eukaryotic pathogens. *Proceedings of the National Academy of Sciences of the United States of America*.

[44] Chen X., Xu L., Jin L. (2016). Efficient and regioselective synthesis of *β*-galNAc/GlcNAc-lactose by a bifunctional transglycosylating *β*-N-acetylhexosaminidase from *Bifidobacterium bifidum*. *Applied and Environmental Microbiology*.

[45] Ando S., Kon K., Nagai Y., Yamakawa T. (1982). A novel pentaglycosyl ceramide containing di-beta-N-acetylgalactos-aminyl residue (Para-Forssman glycolipid) isolated from human erythrocyte membrane. *Advances in Experimental Medicine and Biology*.

[46] Gerwig G. J., van Kuik J. A., Leeflang B. R. (2002). The *Giardia intestinalis* filamentous cyst wall contains a novel *β*(1-3)-*N*-acetyl-D-galactosamine polymer: a structural and conformational study. *Glycobiology*.

[47] Thorn J. J., Levery S. B., Salyan M. E. K. (1992). Structural characterization of x2 glycosphingolipid, its extended form, and its sialosyl derivatives: accumulation associated with the rare blood group p phenotype. *Biochemistry*.

[48] Cunningham M. W. (2012). Streptococcus and rheumatic fever. *Current Opinion in Rheumatology*.

[49] Briko N. I., Bovin N. V., Shevelev B. I. (1997). Immunoenzyme test system for detecting antibodies to group-specific antigens of group A Streptococcus on the base of conjugated N-acetylglucosamine and its use in medical practice. *Klinicheskaia Laboratornaia Diagnostika*.

[50] Chen Y., Yang F., Lu H. (2011). Characterization of fecal microbial communities in patients with liver cirrhosis. *Hepatology*.

[51] Hamadeh R. M., Jarvis G. A., Galili U., Mandrell R. E., Zhou P., Griffiss J. M. (1992). Human natural anti-Gal IgG regulates alternative complement pathway activation on bacterial surfaces. *The Journal of Clinical Investigation*.

[52] Mehta A. S., Long R. E., Comunale M. A. (2008). Increased levels of galactose-deficient anti-Gal immunoglobulin G in the sera of hepatitis C virus-infected individuals with fibrosis and cirrhosis. *Journal of Virology*.

[53] McMorrow I. M., Comrack C. A., Nazarey P. P., Sachs D. H., DerSimonian H. (1997). Relationship between abo blood group and levels of gal *α*,3galactose-reactive human immunoglobulin G1. *Transplantation*.

[54] Kalambokis G., Kolios G., Seferiadis K., Tsianos E. V. (2005). Serum oligoclonal immunoglobulin bands in cirrhotic patients. *The American Journal of Gastroenterology*.

[55] Raska M., Takahashi K., Czernekova L. (2010). Glycosylation patterns of HIV-1 gp 120 depend on the type of expressing cells and affect antibody recognition. *The Journal of Biological Chemistry*.

[56] Cooling L. (2015). Blood groups in infection and host susceptibility. *Clinical Microbiology Reviews*.

[57] Campbell C. T., Gulley J. L., Oyelaran O., Hodge J. W., Schlom J., Gildersleeve J. C. (2014). Humoral response to a viral glycan correlates with survival on PROSTVAC-VF. *Proceedings of the National Academy of Sciences of the United States of America*.

[58] Bilski J., Mazur-Bialy A., Wojcik D. (2017). The role of intestinal alkaline phosphatase in inflammatory disorders of gastrointestinal tract. *Mediators of Inflammation*.

[59] Pike A. F., Kramer N. I., Blaauboer B. J., Seinen W., Brands R. (2013). A novel hypothesis for an alkaline phosphatase ‘rescue’ mechanism in the hepatic acute phase immune response. *Biochimica et Biophysica Acta*.

[60] Kovarik P., Castiglia V., Ivin M., Ebner F. (2016). Type I interferons in bacterial infections: a balancing act. *Frontiers in Immunology*.

[61] Sleijfer S., Bannink M., Gool A. R., Kruit W. H. J., Stoter G. (2005). Side effects of interferon-*α* therapy. *Pharmacy World & Science*.

[62] Kato-Motozaki Y., Komai K., Takahashi K. (2009). Polyethylene glycol interferon alpha-2b-induced immune-mediated polyradiculoneuropathy. *Internal Medicine*.

